# Reparability of giomer using different mechanical surface treatments

**DOI:** 10.4317/jced.53591

**Published:** 2017-04-01

**Authors:** Saba Arami, Soodabeh Kimyai, Parnian-Alizadeh Oskoee, Mehdi Daneshpooy, Sahand Rikhtegaran, Mahmoud Bahari, Mehdi-Abed Kahnamoii

**Affiliations:** 1 Post graduate student, Department of Operative Dentistry, Faculty of Dentistry, Tabriz University of Medical Sciences, Tabriz, Iran; 2 Dental and Periodontal Research Center, Faculty of Dentistry, Tabriz University of Medical Sciences, Tabriz, Iran; 3 Professor, Department of Operative Dentistry, Faculty of Dentistry, Tabriz University of Medical Sciences, Tabriz, Iran; 4 Assistant Professor, Department of Operative Dentistry, Faculty of Dentistry, Tabriz University of Medical Sciences, Tabriz, Iran; 5 Associate Professor, Department of Operative Dentistry, Faculty of Dentistry, Tabriz University of Medical Sciences, Tabriz, Iran

## Abstract

**Background:**

In the repair process achieving high bond strength between the new and old resin based materials is necessary for clinical longevity. This study compared the effect of three different mechanical surface treatments (air abrasion, Nd:YAG laser and diamond bur) on the repair bond strength of giomer.

**Material and Methods:**

In this *in vitro*study, 125 cylindrical giomer samples were used. The giomer samples were randomly assigned to 5 groups (n=25). In group 1, the samples did not undergo any surface treatment. In groups 2 to 4, the samples underwent surface treatments with air abrasion, Nd:YAG laser and a diamond bur. The samples in group 5 were prepared to measure giomer cohesive strength. Subsequently, the new giomer was bonded to the existing giomer in groups 1 to 4. Then the repair bond strength of the samples was measured. One-way ANOVA and post hoc Tukey test were used to compare the bond strength.

**Results:**

There were significant differences between the different surface treatments (*P*<0.001); the repair bond strength in the air abrasion group was significantly higher than that in the Nd:YAG laser group, in which it was significantly higher than that in the diamond bur group, which was in turn higher than that in group 1 (no surface treatment) (*P*<0.001). In addition, the cohesive strength of giomer was significantly higher than the repair bond strength in the 4 other study groups (*P*<0.001).

**Conclusions:**

Of all the surface treatments, air abrasion and Nd:YAG laser, in descending order, yielded the highest repair bond strength values, with the repair bond strength values of 60‒70% of the giomer cohesive strength.

** Key words:**Air abrasion, bond strength, giomer, Nd:YAG laser, repair, surface treatment.

## Introduction

Composite resins are materials with widespread use in direct restorative procedures. The majority of composite resin restorations undergo defects such as abrasion, fatigue or discoloration after some service due to mechanisms such as mechanical and physical degradations, because of contact with water or physical processes such as enzymatic, hydrolytic and acidic reactions or temperature degradation. Therefore, replacement or repair of these restorations has become one of the routine dental procedures (1-4). Repair is more conservative compared to the replacement of restorations and can increase the longevity of restorations at a lower cost (5). In addition, it might prevent pulpal irritation (6,7) and detrimental changes in odontoblasts (1).

In the repair process, it is necessary to achieve a high bond strength between the new and old composite resins for clinical longevity (1,3). It is essential to prepare the surface of the old composite resin in order to remove its surface layer so that a clean surface with high surface energy and a larger surface area can be achieved for bonding through surface roughening (8). In order to improve bonding during the repair process, different mechanical and chemical surface treatments have been suggested, including use of burs, air abrasion, different lasers, covering the bonding surface with silica, use of hydrofluoric acid, phosphoric acid, silane and finally the use of bonding agents (7-9). Different studies have compared the effects of various surface treatments on the repair bond strength of different composite resins, with different results being reported depending on the type of substrate and preparation technique (5,8,10-13). Recently, a new group of composite resins has been introduced for direct adhesive restorations, referred to as giomers, with both the advantages of glass-ionomers (release of fluoride and recharging capability) and composite resins (esthetic appearance, easy polishability and biocompatibility) (14). A 13-year clinical trial showed that the majority of giomer restorations exhibited favorable clinical quality during the recall visits (15). Since no study to date has evaluated the effects of different mechanical surface treatments on the repair bond strength of giomer restorations, the aim of the present study was to evaluate the effects of three different mechanical surface treatments (air abrasion, Nd:YAG laser and a diamond bur) on the repair bond strength of giomer.

## Material and Methods

The present *in vitro* study was carried out on 125 cylindrical giomer samples. The protocol of the study was approved by Regional Medical Research Ethics Committee. Giomer (Beautifil II; Shofu Inc., Kyoto, Japan) samples were prepared by placing 2-mm layers of giomer in plastic molds in groups 1 to 4, which measured 4 mm in height and 6 mm in diameter and in group 5, 6 mm in height and 6 mm in diameter. Each giomer layer was light-cured for 20 seconds with the use of an Astralis 7 (Ivoclar Vivadent, AG, FL-9494, Schaann, Liechtenstein) light-curing unit according to manufacturer’s instructions. The last layer was covered with a piece of translucent matrix band (Have Neos Dental, Bioggio, Switzerland) and compressed with a glass slide to achieve a smooth surface before it was light-cured. After light-curing, the samples were retrieved from the molds and light-cured again for 40 seconds with the use of Astralis 7 light-curing unit, and then placed in acrylic blocks with 2 mm of the cylinders within the acrylic blocks. The samples were incubated in distilled water at 37°C for 3 weeks (16). Then the samples were divided into 5 groups (n=25) as follows:

Group 1 (negative control): No giomer surface preparation was carried out in this group, FL-Bond II (Shofu Inc., Kyoto, Japan) self-etch adhesive system was applied on the giomer surface according to manufacturer’s instructions. A new layer of giomer, 2 mm in thickness (two 1-mm layers, each layer light-cured for 20 seconds with the use of Astralis 7 light-curing unit), was bonded to the surface of the existing giomer and light-cured at a light intensity of 400 mW/cm2 at a right angle to the surface and barely touching the giomer surface. It should be pointed out that in order to add the new giomer to the old one, a plastic mold, measuring 2 mm in height and 4 mm in diameter, was placed at the center of the old giomer and then the new giomer was packed within it. Then the samples were retrieved from the molds and light-cured again for 40 seconds with Astralis 7 light-curing unit. Then all the samples were incubated in distilled water at 37°C for 24 hours. The repair bond strength of the samples was determined with the use of Hounsfield Test Equipment (Model HSK-S, Salfords, Redhill, Surrey, England) at a crosshead speed of 1 mm/min. The samples were fractured by the chisel-shaped blade of the equipment at old giomer‒new giomer interface and the bond strength in megapascal was calculated by dividing the forced applied (in newton) by the bonding surface area (in square millimeter). After fracturing the samples, the fracture modes were classified as follows:

Adhesive fracture: fracture at old giomer-new giomer interface

Cohesive fracture: fracture within the old or new giomer

Mixed fracture: a combination of the two above 

Group 2: All the procedures were similar to those in group 1, except for the fact that in this group, first the giomer surface was roughened with an air abrasion equipment (Microblaster Dento-Prep TM, Dental Microblaster, Denmark) using 50-µ aluminum oxide particles under a pressure of 50 bar (60 PSI) with the equipment tool 5 mm away from the sample surface at a right angle to it for 10 seconds.

Group 3: All the procedures were similar to those in group 1, except for the fact that in this group first the giomer surface was roughened with Nd:YAG laser (Nd:YAG Dental Laser, LAMBDA, Scientific S.r.l., Vicenza, Italy) at a distance of 2 cm at a right angle to the surface for 10 seconds using the following parameters: frequency=20 Hz, power=3 W, energy=150 mJ and fiber diameter=400 µm).

Group 4: All the procedures were similar to those in group 1 except for the fact that in this group, the giomer surface was roughened with a coarse (001) diamond fissure bur (Diatech Dental AG, Swiss Dental Instruments, CH-9435 Heerbrugg) with particle sizes measuring 125-150 µm, in a high-speed handpiece under water spray for 3 seconds. The bur was held tangential to the giomer surface and one bur was used for every 5 samples.

Group 5 (positive control): The giomer samples were placed in Hounsfield Test Equipment to determine the cohesive strength without adding new giomer.

Then the surfaces of the samples in groups 2-4 (with mechanical surface treatments) were cleaned in an ultrasonic bath for 10 minutes. In order to evaluate the surface ultrastructure and topography after mechanical surface treatments, two extra samples of giomer in each group (without surface treatment, treatment with air abrasion, treatment with Nd:YAG laser and treatment with a diamond bur [with the use of no adhesive and new giomer on the old giomer]) were prepared and their ultrastructure was evaluated under scanning electron microscope (SEM) at ×500 (CamScan MV2300, Brno, Czech Republic); the surface topography of these samples was evaluated under an atomic force microscope (AFM) (Nano Scope ® II, Digital Instruments, USA). A silica nitride tip, measuring 50 nm in radius, with an apex angle of 45°, connected to a fixed substrate on a cantilever, was utilized for AFM analysis procedures. The image had a resolution of 256×256 pixels, and the scan rate was adjusted at 1.9 Hz. Scanning was carried out on the surface of the specimens in quadrants, consisting of 10×10-μm areas.

Data were evaluated with descriptive statistics (means and standard deviations) with SPSS (version 20.0, SPSS, Chicago, IL, USA). Kolmogorov-Smirnov test was used to evaluate normal distribution of data; Levene’s test was used to evaluate equality of variances. One-way ANOVA was used to compare the bond strength between the groups; and post hoc Tukey test was used for two-by-two comparisons of the groups. Statistical significance was set at *P*<0.05.

## Results

**Figure 1:**
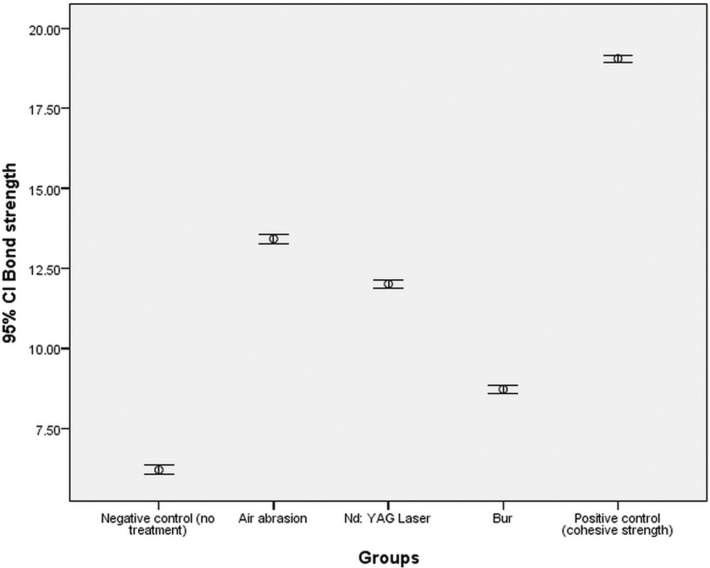
Error-bar graph of mean bond strength values in the study groups.

 Table 1 shows descriptive statistics of bond strengths and statistical comparisons between the study groups. Figure 1 shows the error-bar graph of mean bond strength values in the study groups. Evaluation of the results of one-way ANOVA showed that the mean bond strength values were significantly different from each other in terms of mechanical surface treatments (F4,120=6152.101, *P*<0.001). In addition, there were significant differences between the different mechanical surface treatments based on the results of post hoc Tukey test (*P*<0.001). The repair bond strengths in different groups in descending order were as follows: air abrasion>Nd:YAG laser>diamond bur>negative control group (P<0.001). In addition, the cohesive strength of giomer was significantly higher than the repair bond strengths in the other 4 groups (*P*<0.001).

**Table 1:**
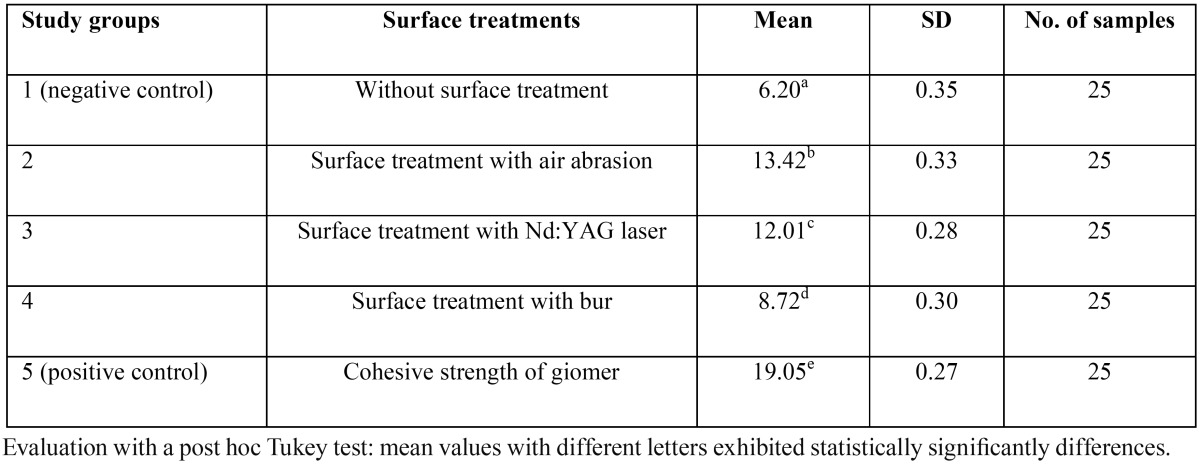
Means and standard deviations (SD) of repair bond strength values (MPa) in the study groups.

 Table 2 presents the frequencies of fracture modes. Cohesive fracture was seen in the positive control samples only. In the negative control and the diamond bur groups the fractures were predominantly of the adhesive type, while in the air abrasion and Nd:YAG laser groups the fractures were mainly of the mixed type.

**Table 2:**
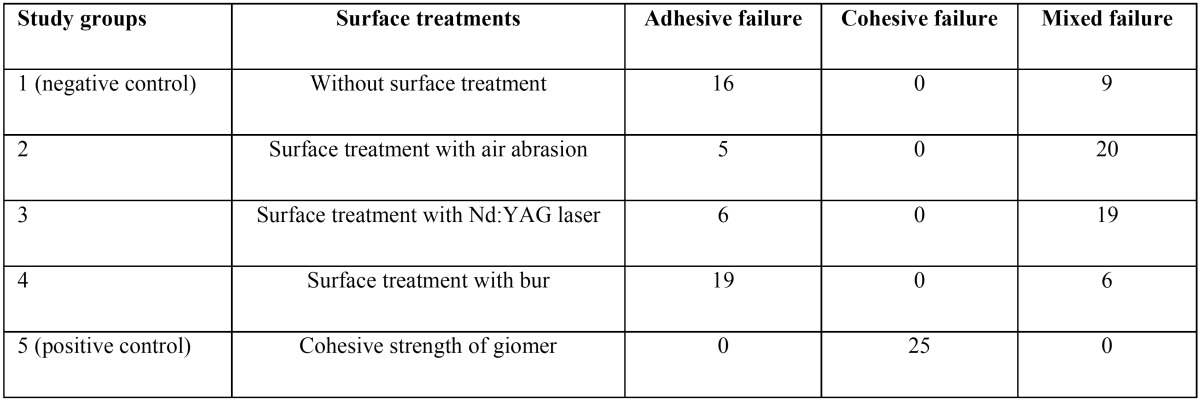
The frequencies of fracture modes in the study groups.

**Figure 2:**
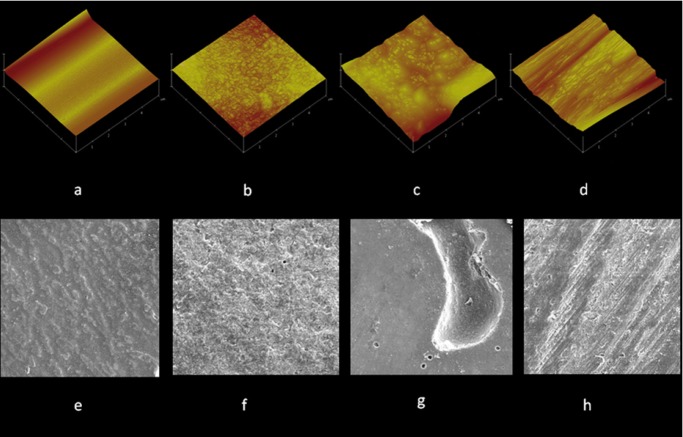
Scanning electron microscope (×500) and atomic force microscope micrographs of giomer samples in the study groups: a and e, with no mechanical surface treatment; b and f, mechanical surface treatment with air abrasion; c and g, mechanical surface treatment with Nd:YAG laser; and d and h, mechanical surface treatment with the use of diamond bur.

As shown in figure 2, the SEM and AFM images in the negative control group exhibited a smoother surface compared to the other groups. However, in the samples in the air abrasion group, homogeneous and fine irregularities were diffusely seen on the surface. In samples prepared with Nd:YAG laser, deep and large cavities were visible on the surface. In samples prepared with a diamond bar, sharp surface irregularities were created and microretentive features were seen on the surface in a linear pattern.

## Discussion

Achieving high bond strength between the old giomer and the new giomer that is added during the repair process is an important factor in the success of the repair process. The results of the present study showed that the repair bond strength of giomer after mechanical surface treatments (air abrasion, Nd:YAG laser and diamond bur) increased significantly, which is consistent with the results of previous studies on resin-based materials, indicating that mechanical roughening of the substrate surface is an important factor in increasing the repair bond strength (9-11,17). An increase in repair bond strength might be attributed to an increase in surface roughness of the substrate and an increase in bonding surface, as confirmed by SEM and AFM images. The negative control group samples exhibited more smooth surfaces compared to other groups, which might explain the lower repair bond strength in this group. In addition, it has been shown that subsequent to roughening, the surface energy and wettability of the adhesive agent increase (5,11,12).

It has been reported that irradiation of the surface of a composite resin with Nd:YAG laser leads to the dispersion of the laser energy by silica filler particles; an increase in the absorption of this energy by the resin component of composite resin gives rise to localization of this energy near the substrate surface, modifying the heat of the composite matrix, which finally results in its removal to produce surface irregularities (12). Giomers use pre-reacted glass filler technology, in which pre-reaction of fluroaluminosilicate glass fillers with polyacrylic acid results in the formation of a stable phase called “wet siliceous hydrogel”, which is then freeze-dried, milled, silanized and ground to produce PRG fillers. Beautifil II uses S-PRG (surface reaction type), in which polyacrylic acid attacks only the surface of the glass filler, leaving a glass core (18). Since giomer is a biphasic material, consisting of a resin matrix and filler particles, it is possible to believe that the mechanism of the effect of laser on composite resins holds true in the case of giomer, too. In this context, previous studies have shown that surface roughening with Nd:YAG laser results in an increase in repair bond strength of indirect (12) and silorane-based composite resins (8). However, in a study by Akyil *et al.*, use of Nd:YAG laser alone did not result in an increase in repair bond strength of feldspathic ceramic (19). The discrepancy between the results might be attributed to differences in substrates (composite resin or ceramic) and their different response to laser and also the differences in laser parameters.

It has been reported that air abrasion increases the repair bond strength of composite resins by creating larger microretentive areas, increasing surface roughness, increasing substrate surface, decreasing surface tension and increasing wettability (11,20). SEM and AFM images in the present study confirmed this. Previous studies (9,12,21) have shown that roughening the surface with air abrasion results in an increase in the repair bond strength of composite resins. Contrary to the results of the present study, Alizadeh *et al.* (10) reported that use of air abrasion did not result in an increase in the repair bond strength of silorane-based composite resin. This decrease has generally been attributed to the exposure of filer particles after abrasion, decreasing the availability of the resin for primary bonding. In addition, it has been reported that the interference of surface debris with the repair process and the presence of air inclusion at the interface decreases the surface available for bonding (10). It appears the different nature of substrates (giomer and silorane-based composite resin) and interference of debris on the surface after preparation with air abrasion, as a result of not using an ultrasonic device to clean the surface in the previous study (10), might be a reason for differences in the results of the present study and the study above.

It has been shown that use of bur increases the repair bond strength of composite resins by roughening the substrate surface (10,11,22). SEM and AFM images in the present study confirmed roughening of the substrate surface and explained the increased repair bond strength after diamond bur surface preparation. Contrary to this study, Palasuk *et al.* reported that use of a diamond bur did not result in a significant increase in repair bond strength of silorane-based composite resin (9). In addition, in a study by Loomans *et al.*, there was no significant difference in repair bond strength of heavily hybrid Clearfil PhotoPosterior composite resin between the control group (with no surface preparation) and the group prepared with a diamond bur (4). The differences in the results might be attributed to not using an ultrasonic device after preparation of the surface with a diamond bur and interference of the debris in the smear layer with the bonding process, resulting in a decrease in repair bond strength. A difference in the type of the composite resin used might be another reason for differences in the results. It has been reported that the type of the composite resin has an important role in the repair bond strength because different composite resins react differently to different techniques of repair (4,9).

Another finding of the present study was the higher repair bond strength in the air abrasion group compared to the Nd:YAG laser group, consistent with the results of previous studies on indirect composite resins (12) and ceramics (23). In addition, in another study, air abrasion technique resulted in higher repair bond strength in indirect composite resins compared to Er,Cr:YSGG laser (24). The lower repair bond strength in the Nd:YAG laser group compared to the air abrasion group might be explained by destruction of the matrix and the crystalline phase and separation of these two phases in association with the formation of deep undercuts. In SEM images, Nd:YAG laser resulted in the creation of deep cavities on the giomer surface, which might result in subsurface destruction, compromising the bond. In a previous study on indirect composite resin, the use of Nd:YAG laser resulted in deep cracks on the surface (12). Contrary to the results of the present study, in a study on silorane-based composite resin, preparation of the surface with Er,Cr:YSGG laser resulted in a significant increase in repair bond strength compared to preparation of the surface with air abrasion (10). In addition, in another study on laboratory composite resin, Er,Cr:YSGG laser resulted in repair bond strength similar to that with the use of air abrasion (11). The differences in the results of the studies above and the present study might be attributed to the type of the laser and different performances of the lasers used.

Based on the results of the present study, use of air abrasion and Nd:YAG laser resulted in higher repair bond strength compared to the diamond bur group. In the previous study, the use of air abrasion resulted in a higher repair bond strength compared to the use of a diamond bur (17). It has been postulated that different mechanical surface treatments might result in differences in smearing and matrix cracking, affecting the bond strength. It has been demonstrated that the smear layer has a negative effect on the bond of adhesive resins due to its lower surface energy (11). Air abrasion creates microretentive and a diamond bur crates micro- and macroretentive features (17). It has been reported that in the presence of the bonding agent the bond strength on the surface prepared with air abrasion is higher than that on surface prepared with a diamond bur due to its infiltration into the microscopic irregularities (11). Contrary to the results of the present study, in a previous study, preparation of the surface of direct composite resin with the use of a diamond bur resulted in higher repair bond strength compared to air abrasion (22). In addition, another study did not show any significant difference in the repair bond strength of direct composite resin between the two above-mentioned surface preparation techniques (25). The differences in the results might be attributed to differences in the substrates and use of different adhesive systems because the surface characteristics and composition of the substrate can affect the efficacy of the mechanical surface treatments (4,11). In addition, the surface tension and viscosity of the adhesives can affect their penetration into the surface irregularities (11).

In this study, only in two groups of Nd:YAG laser and air abrasion the mean repair bond strength reached almost 60-70% of the cohesive strength of giomer, which was considered clinically acceptable for composite resins based on previous studies (8,10). In a silorane-based composite resin with the use of Nd:YAG laser similar results were achieved (8). However, in a different study on silorane-based composite resin, it was shown that surface preparation with a bur and Er,Cr:YSGG laser resulted in a repair bond strength of approximately 70% of the cohesive strength, while surface preparation with air abrasion did not result in a repair bond strength of approximately 60-70% of the cohesive strength (10).

Based on the results of the present study, the fracture mode of the majority of the samples in the groups prepared with laser and air abrasion was mixed; the fracture mode in the diamond bur and negative control groups was mainly of the adhesive type and cohesive fracture was only seen in the positive control group. A study on indirect composite resins showed that the fracture mode in the groups prepared by air abrasion and Nd:YAG laser was mainly mixed and in the no preparation group it was mainly of the adhesive type (12). Another study on laboratory composite resin showed that in air abrasion and Er,Cr:YSGG groups the fracture mode was mainly mixed, while in the diamond bur and no surface preparation groups the majority of the fractures were adhesive (11). However, in another study no significant differences were observed in fracture modes between different surface preparation techniques (i.e. use of hydrofluoric acid, diamond bur, sandblasting with aluminum oxide particles and covering the surface with silica) with a silorane-based composite resin (26). Differences in the type of substrate, the adhesive resin and different surface treatments might explain differences between the results of different studies.

Since the lasers parameters affect their rate of penetration and ablation, it is suggested that in future studies different parameters of Nd:YAG laser and also other lasers be used for the evaluation of repair bond strength of giomer. Because the age of the restoration to be repaired has an important role in the repair bond strength and since various changes take place during the aging process, including water sorption, chemical decomposition, crack formation and resin‒filler debonding in composite resins (4), it is suggested that different aging protocols be used in association with load cycling and thermocycling with different cycles in future studies. In addition, future long-term clinical studies are recommended to evaluate bonding durability.

Under the limitations of the present study, it might be concluded that different surface treatments result in an increase in repair bond strength of giomer compared to group in which no mechanical surface treatment was applied. The highest repair bond strength values were recorded in the air abrasion, Nd:YAG laser and diamond bur groups, respectively. However, only in the air abrasion and Nd:YAG laser groups the repair bond strength reached 60‒70% of the cohesive strength of giomer.
